# Selenium preserves keratinocyte stemness and delays senescence by maintaining epidermal adhesion

**DOI:** 10.18632/aging.101322

**Published:** 2017-11-25

**Authors:** Lara Jobeili, Patricia Rousselle, David Béal, Eric Blouin, Anne-Marie Roussel, Odile Damour, Walid Rachidi

**Affiliations:** ^1^ Cell and Tissue Bank of Hospices Civils de Lyon, Hôpital Edouard Herriot, Lyon, France; ^2^ Laboratoire de Biologie Tissulaire et Ingénierie Thérapeutique, UMR 5305, CNRS, University Lyon 1, Lyon, France; ^3^ CarMeN Laboratory, INSERM U-1060, INRA USC-1235, Lyon 1 University, Lyon, France; ^4^ Grenoble Alpes University, Grenoble, France; ^5^ CEA, INAC, SyMMES, Grenoble, France; ^6^ Labcatal Pharmaceuticals, Montrouge, France

**Keywords:** selenium, replicative life span, skin aging, adhesion, keratinocytes stem cells

## Abstract

Skin is constantly exposed to environmental factors such as pollutants, chemicals and ultra violet radiation (UV), which can induce premature skin aging and increase the risk of skin cancer. One strategy to reduce the effect of oxidative stress produced by environmental exposure is the application of antioxidant molecules. Among the endogenous antioxidants, selenoproteins play a key role in antioxidant defense and in maintaining a reduced cellular environment. Selenium, essential for the activity of selenoproteins, is a trace element that is not synthesized by organisms and must be supplied by diet or supplementation. The aim of this study is to evaluate the effect of Selenium supplementation on skin aging, especially on keratinocytes, the main cells of the epidermis. Our results demonstrate for the first time to our knowledge, the major role of Selenium on the replicative life span of keratinocytes and on aging skin. Selenium protects keratinocyte stem cells (KSCs) against senescence via preservation of their stemness phenotype through adhesion to the basement membrane. Additionally, Selenium supplementation maintains the homeostasis of skin during chronological aging in our senescent skin equivalent model. Controlled supplementation with Selenium could be a new strategy to protect skin against aging.

## INTRODUCTION

The skin, like every organ and tissue of the human body, is prone to aging. However, the skin aging process is affected by both intrinsic and extrinsic factors. Skin is composed of a pluristratified epidermis firmly anchored to the dermis through a complex structure, the dermal epidermal junction (DEJ). Aging impacts both the epidermal and dermal parts of the skin, with a progressive loss of homeostasis, especially in the balance between proliferation and differentiation of the epidermis [1] and in the loss of interaction between the dermis and epidermis via disorganization of the DEJ [2]. Keratinocytes are the main cells of the epidermis and exist at various differentiation states from the basal layer, which is the only proliferative layer, to the non-living layer, the stratum corneum. Keratinocyte stem cells (KSCs) are necessary to ensure constant renewal of the epidermis throughout life. They are maintained and protected as stem cells in a microenvironment called a “niche” and are strongly anchored to the DEJ through β1 and α6 integrin binding to type IV collagen and laminin 332, respectively, the main components of the basement membrane [3,4]. To differentiate, KSCs break their interactions with the basement membrane and migrate to the suprabasal layers of the epidermis. KSCs interactions with the DEJ are therefore crucial for stemness, homeostasis, and skin structural integrity.

To date, photoaging is more studied than chronologic aging because skin is constantly exposed to several oxidative environmental stressors (e.g., ultra violet radiation (UVA and UVB), natural ionizing radiation, pollutants, and chemicals) that contribute to pre-mature skin aging signs such as pigmentary stains, deep wrinkles, and an increased risk of skin cancer [5,6]. Thus, enhancement of the endogenous and/or exogenous antioxidant defenses could be a beneficial strategy to fight the effects of photoaging. Among the endogenous antioxidants, selenoproteins, which require the essential trace element Selenium for their activity, are the most important enzymes that participate in the protection of the entire organism against oxidative stress, with skin as the special target [7,8]. Several groups have shown that Selenium supplementation protect keratinocytes [9,10,11] melanocytes [12] and fibroblasts [13,14] from UV-induced cell death and DNA damage. Few articles have reported the importance of Selenium and selenoproteins on skin homeostasis in animals or humans. As described by Bates *et al*., Selenium-deficient rats display a slower growth rate and sparse hair growth [15]. Additionally, the case of a young child with severe Selenium deficiency due to long-term parenteral nutrition was highlighted. This young child had dry skin and erythematous changes associated with cardiomyopathy [16]. An oral supplementation with Selenium resulted in a complete disappearance of the lesions and defects. These two studies reported cutaneous manifestations of Selenium deficiency. Sengupta *et al*. went further by implementing stable inactivation of selenoproteins in K14-expressing epidermal cells in a mouse model [17]. This led to abnormalities in skin, such as a decrease in epidermal thickness, wrinkle formation and epidermal detachment focally along the DEJ. Moreover, keratinocytes extracted from those mice showed an altered morphology and a lack of adhesion under standard culture conditions. These results demonstrate a link between selenoprotein expression and the maintenance of skin homeostasis. Moreover, it has been reported that replicative senescence of human fibroblast WI38 cells is controlled by Selenium levels and that this senescence selectively modulates selenoprotein expression [18]. Selenoproteins, especially seleno-protein H, are important in genomic integrity and the preservation of cells from senescence [19]. The correlations among serum Selenium concentration, activity of selenoproteins, age, and longevity have been described, with a decrease in serum Selenium with age in healthy subjects, especially those over 60 years old [20]. In addition, low serum Selenium level is an important predictor of shortened longevity in elderly patients [20,21]. These studies indicate an increased requirement of Selenium supplementation in elderly persons.

The objective of this study was to investigate the effect of Selenium supplementation on chronological aging. First, we selected the lowest dose shown to have a positive effect on keratinocyte viability and clonogenic potential over replicative life span. Second, we tested the impact of Selenium supplementation in a 3D skin aging model [22,23] during an extended culture time [24]. This research focuses on the impact of Selenium on skin aging and highlights that Selenium can preserve KSCs in vitro via activation of adhesive properties throughout the cells' replicative life span and delay senescence of the epidermis in an extended live-culture skin model.

## RESULTS

### Effect of Sodium Selenite supplementation on keratinocyte viability and clonogenic potential

**Figure 1:**
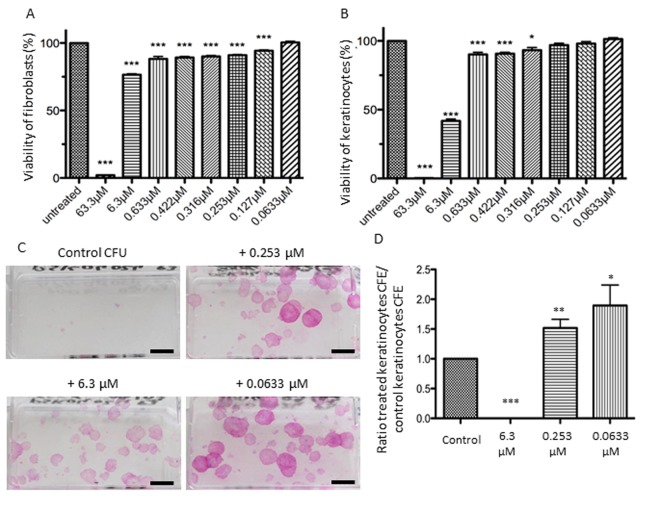
Influence of selenium on cell viability and proliferation Effects on cell viability after application of a range of NaSe concentrations during 72 h for (**A**) fibroblasts and (**B**) keratinocytes. Viability is calculated as the “(mean of treated cells–mean of blank) / (mean of untreated cells–mean of blank)” ±SD (n=3 donors and n=12 measurements per conditions). The stars illustrate statistical differences between 100 % and mean of experimental points. Effects of 3 Selenium concentrations on clonogenic potential of keratinocytes from 3 donors after 12 days of supplementation. (**C**) Representative aspect of the colonies at different concentrations versus the control, (n=3; scale bar=1cm) and (**D**) CFE ratio are calculated to avoid inter-individual heterogeneity of CFE. *p<0.05; **p<0.01; ***p<0.001.

The effects of different concentrations of NaSe on cell viability were assessed using an MTT test on fibroblasts and keratinocytes (Figure 1A and B, respectively). Concerning fibroblasts, the first concentration that shows no significant toxicity is 0.0633 μM. The highest concentration tested (63.3 μM) is highly cytotoxic with 2% cell viability, but between 6.3 μM and 0.127 μM, cell viability ranges from 76% to 94% (***p<0.001). Concerning keratinocytes, they are more sensitive to the strong dose of NaSe, and at 63.3 μM there is no cell viability. Additionally, between 6.3 μM and 0.316 μM, the cell viability ranges from 40% to 94% (***p<0.001 and *p<0.05, respectively). Then, the effect of 0.253 μM and 0.127 μM of NaSe allows cell viability of approximately 98%, and the cytotoxic effect is not significant. One hundred percent viability is reached with the lowest dose (0.0633 μM). Interestingly, keratinocytes are also more resistant to the low dose of NaSe compared to fibroblasts.

Then, we performed a clonogenic assay with three selected concentrations of NaSe: 6.3 μM, 0.253 μM and 0.0633 μM. With a long-term exposure of low-density keratinocytes to NaSe, the results are comparable to short-term exposure. No clones are observed with 6.3 μM, confirming the toxic concentration, whereas for both concentrations (0.253 μM and 0.0633 μM), we observed a significant increase in the number of holoclones compared to the control condition (Figure 1C). The number of holoclones allows for the calculation of CFE (colony forming efficiency), clearly demonstrating that NaSe can improve the CFE ratio of keratinocytes from 1.5 to 2 times (Figure 1D **p<0.01 and *p<0.05).

Those results prompted us to explore lower concentrations of NaSe to obtain the lowest dose possible to maximize the effect on keratinocytes. We selected 8 concentrations in a range of from 0.633 μM to 1.875 nM.

**Figure 2:**
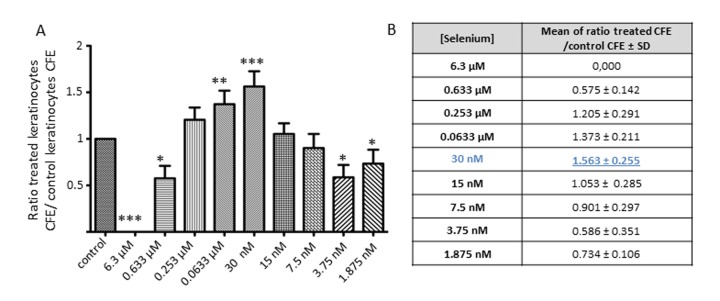
Effect of a low dose of Selenium on keratinocyte clonogenic potential (**A**) Average of the CFE ratio after treatment (KT CFE) versus control KC CFE (KT CFE/KC CFE) obtained after 12 days of Selenium application over a range of concentrations from 6.3 μM to 1.875 nM, and (**B**) results are expressed as the mean ± SD of 3 donors (n=3). CFE ratios are calculated to avoid inter-individual heterogeneity of CFE. *p<0.05; **p<0.01; ***p<0.001.

Figure 2 shows the CFE results obtained after the treatment of keratinocytes from 3 independent donors with these selected concentrations. Because of the well-known inter-individual variation and strong variation of CFE, the results are expressed as the ratio of the treated keratinocyte CFE to the control keratinocyte CFE. The ratio is significantly increased in the range from 0.253 μM to 30 nM. Regardless of the donor, the concentration of 30 nM NaSe leads to the highest CFE ratio of 1.563 ± 0.255 (***p<0.001).

Since this concentration improves the capacities of keratinocytes to give rise to holoclones, 30 nM NaSe was selected for constant supplementation of the culture medium for all the following experiments.

### Sodium selenite supplementation delays the replicative senescence

**Figure 3:**
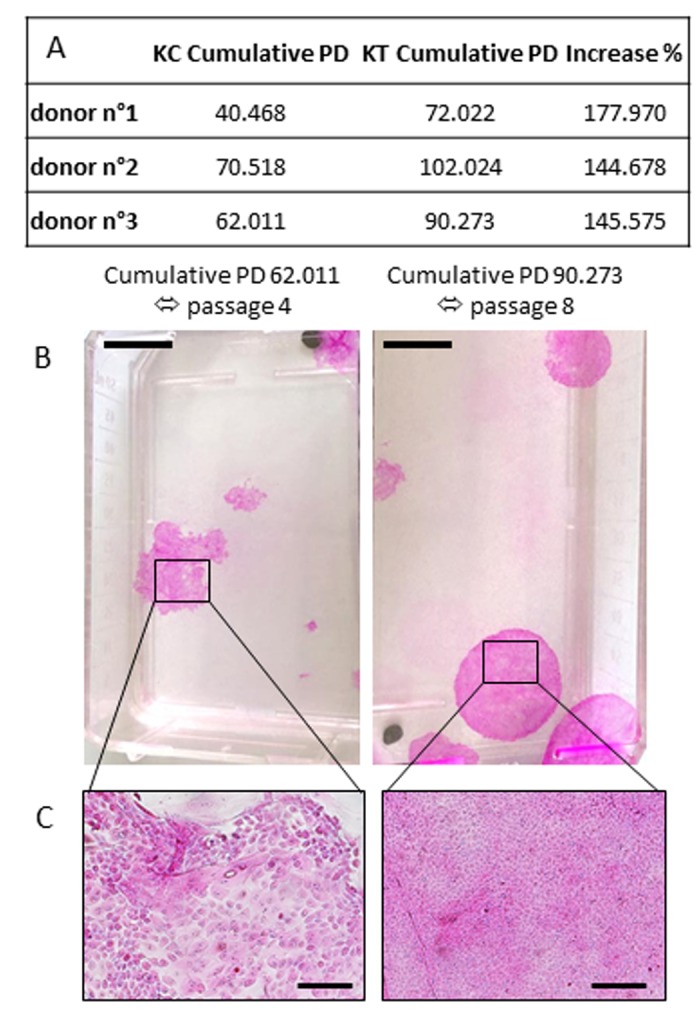
Influence of Selenium on replicative senescence (**A**) Cumulative population doubling corresponds to the sum of population doubling over subculture passages until the last passage with holoclones. The percent increase is calculated as (KT Cumulative PD/KC Cumulative PD) x100 (KT: treated keratinocytes; KC: control keratinocytes). CFE was calculated at each passage over replicative life span where keratinocytes are still able to produce holoclones. (**B**) Example of colony morphology corresponding to the last cumulative PD/passages with holoclones. (Scale bar = 1cm) (**C**) Representative morphology of supplemented keratinocytes (small, cohesive and cobblestone keratinocytes) or control keratinocytes (senescence-associated phenotype with differentiated cells) at a late passage. Representative photographs are shown (scale bar=500 μm; n=3 donors).

The proliferative capacity of primary keratinocytes measured via subculture of the cumulative population doubling (PD; Table A in Figure 3) demonstrates in all cases that NaSe supplementation extended the replicative lifespan of human keratinocytes. Regardless of the donor, the cumulative PD that leads to holoclones appears to always increase by 156.074 ± 14.597 % after 30 nM NaSe supplementation. NaSe treatment supports the presence of holoclones at least until passage 8 despite the donor (corresponding to 88.106 ± 15.118 cumulative PD), whereas in the control group, the holoclones are absent since passage 4 (corresponding to 57.666 ± 15.489 cumulative PD). Associated with the proliferative phenotype of the non-supplemented culture, the morphological aspects of the colonies are dramatically changed, as shown by a loss of smooth shape and a decrease in size, and are composed of differentiated keratinocytes with senescence morphology (Figure 3B and C, left panel).

### Sodium selenite supplementation activates adhesion to type IV collagen and laminin 332 of human primary keratinocytes

**Figure 4:**
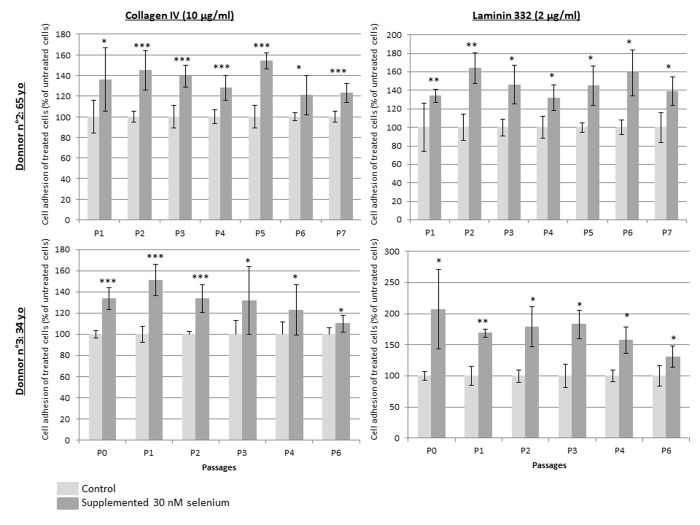
Keratinocyte relative adhesion to type IV collagen or laminin 332 during replicative senescence Keratinocytes from 2 donors over subculture passages with or without SeNa supplementation and seeding at each passage on pre-coated wells with 10 μg/ml type IV collagen (on the left) or with 2 μg/ml laminin 332 (on the right). Results are the mean of n=3 measurements ± SD normalized to the mean of adhesion obtain from poly-D-lysine coated plates. *p<0.025, **p<0.005, ***p<0.0002.

Figure 4 demonstrates that NaSe supplementation improves keratinocyte adhesion during the replicative life span. Regardless of the donor, the passage and the adhesion markers, type IV collagen and laminin 332, which are ligands of β1 integrin and α6 integrin, respectively, the percentage of adhered keratinocytes is significantly increased from 110% to 200% when they are supplemented with NaSe compared to control keratinocytes. Indeed, the increase in adhesion tends to be higher in terms of laminin 332 (157.4% ± 22.9) than in terms of type IV collagen (133.1% ± 12.4). Finally, the adhesion seems to be maximal in the first passages (p1 and p2) and decreases as the subculture continues.

### Sodium selenite supplementation results in delayed skin equivalent senescence

**Figure 5:**
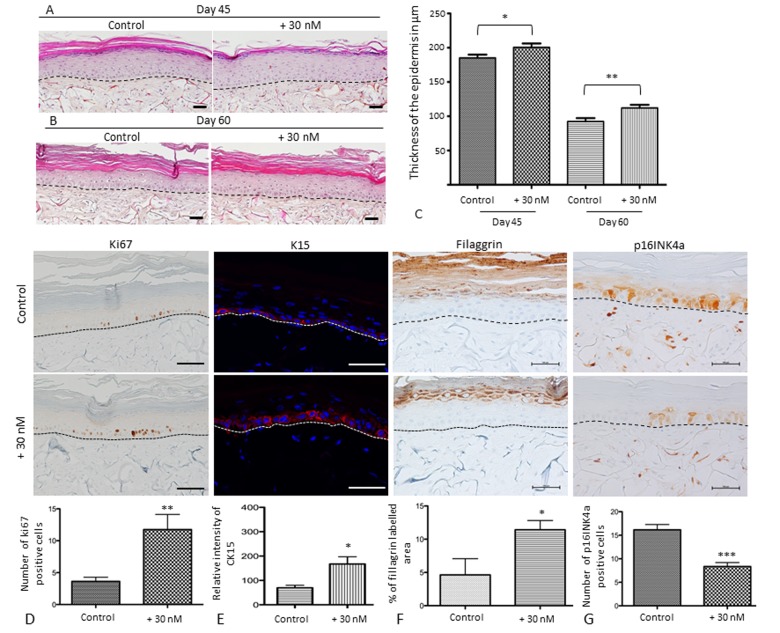
Sodium selenite supplementation improves skin equivalent quality during in vitro senescence and characterization of the balance between proliferation and differentiation of SEs during aging at day 60 Skin equivalents are generated from young keratinocyte and fibroblast donors and cultivated for 45 or 60 days to mimic skin aging [24]. The morphological aspects of SEs that were treated (+30 nM) or not (control) at (**A**) day 45 (D45) corresponding to 17 days of application and (**B**) day 60 (D60) corresponding to 32 days of application. Scale bar = 100 μm (**C**) Quantification of the thickness using ImageJ software is expressed in μm as a distance between from the basal layer of the epidermis to the stratum granulosum excepted for the stratum corneum. (**D**) Immunohistochemical staining of Ki67 in treated (+30 nM) or not (control) SEs and the average number of Ki67-expressing cells (scale bar = 500 μm). (**E**) Immunofluorescence staining of Cytokeratin 15 in treated (+30 nM) or not (control) SEs and the quantification of pixel intensity in relative units. (**F**) Immunohistochemical staining of filaggrin in SEs that were treated (+30 nM) or not (control) and quantification of labeled area in the living epidermis (scale bar = 500 μm). (**G**) Immunohistochemical staining of p16INK4a expression in SEs that were treated (+30 nM) or not (control) and quantification of the number of p16INK4a expressing cells (scale bar = 100 μm). The dermo-epidermal junction is indicated by a dotted line. Results are mean ± SD of 3 independent fields obtained from 3 independent samples. Representative photographs are shown. *p<0.05, **p<0.01.

The histological images of SEs cultured for 45 and 60 days clearly demonstrate that the thickness of the epithelium decreases as the time of culture without treatment increases. A long-term application (17 and 32 days) of 30 nM NaSe supports an SE with a thicker epidermis compared to the control with preservation of terminal differentiation and a decrease in the stratum corneum (Figure 5). At day 45, we can observe a significant increase (108%; *p<0.05) in the epidermis thickness, calculated as 200 μm for the treated SE versus 185 μm for the control SE, and at day 60 we observe an increase of 121% (**p<0.01) in the epidermis thickness, calculated as 112 μm for the treated SE versus 92 μm for the control SE. Moreover, the thickness decreases occur with the time of culture in both the treated and control groups.

Regardless of the time of culture (45 or 60 days), NaSe supplementation allows for the maintenance of SE homeostasis. Therefore, to clarify, Figure 5 D, E, F and G presents the representative results at day 60, which is the time when the senescence is higher. Figure 5D demonstrates a significant increase of 3.2 times in the number of basal keratinocytes expressing the proliferating marker Ki67, which is responsible of epithelium thickness, after NaSe treatment. If we consider the expression of cytokeratin 15 (Figure 5E), a marker of KSCs, the distribution appears to be conserved in the basal layer in both groups. However, the relative intensity of labeling is increased by 2 time in the treated SE compared to the control SE (200 RU and 100 RU, respectively; *p<0.05). These data confirm that NaSe is able to maintain the proliferative capacity of KSCs to ensure the renewal of the epidermis and the maintenance of its thickness during skin aging.

Concerning the late differentiation marker (Figure 5F), the filaggrin expressing area in living epidermis (define as all the layers of the epidermis excepted for the stratum corneum) is 2.5 times higher in the treated SE versus the control SE (approximately 4.6% versus 11.4%, respectively; *p<0.05). These results demonstrate that NaSe preserves the homeostasis of the epidermis with a balance between proliferation and differentiation, with a thicker living epithelium.

Finally, in our SE a p16INK4a is detected in both nuclear and cytoplasmic part of the cells (Figure 5G). NaSe decreases senescence due to long-term culture because the number of cells expressing p16INK4a, a senescence marker, is decreased by 2 times in the treated SE compared to the control SE (***p<0.01). All these data allow us to confirm our hypothesis that NaSe treatment delays SE senescence.

**Figure 6:**
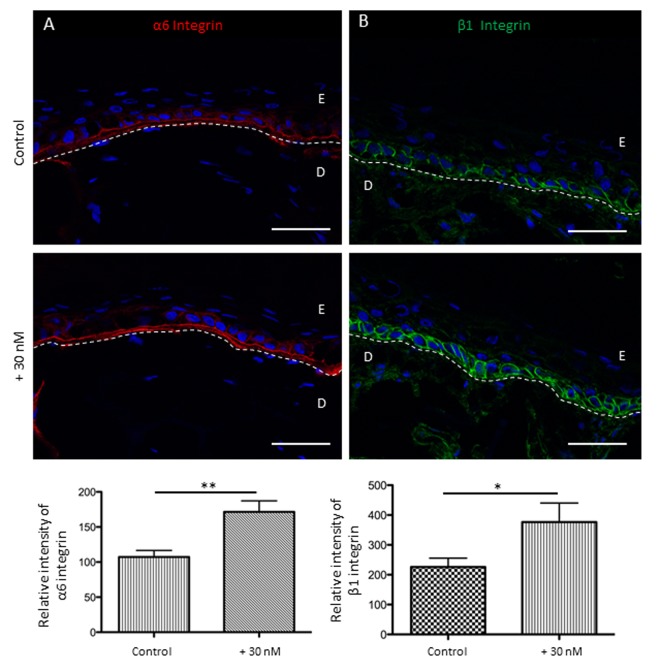
Sodium selenite allows the preservation of keratinocyte adhesion receptors for the basal layer in SEs during aging at day 60 (**A**) Immunofluorescence staining of α6 integrin in SEs that were treated (+30 nM) or not (control), and the quantification is expressed in pixel intensity (relative unit). (**B**) Immunofluorescence staining of β1 integrin in SEs that were treated (+30 nM) or not (control), and the quantification is expressed in pixel intensity (relative unit). Results are the mean ± SD of 5 independent fields from 3 independent samples. Representative photographs are shown; scale bar = 50 μm; E: epidermis; D: dermis. *p<0.05; **p<0.01.

Focusing on adhesion markers, NaSe supplementation during SE development induces the increase of β1 and α6 integrin patterns (Figure 6). The α6 integrin expression appears to be increased by 1.6 times in the treated SE than in the control SE (180.3 ± 65.8 and 101.9 ± 31.5, respectively; **p<0.01). In the same manner, β1 integrin expression is increased by 1.8 times in the treated SE compared to the control SE (376.4 ± 202.2 and 225.9 ± 94.3, respectively; *p<0.05).

## DISCUSSION

Our results show for the first time that a low-dose Selenium supplement has beneficial effects on keratinocyte stemness and delays skin aging. We selected the optimal concentration to include this trace element in our culture condition. In the first experiment, due to inter-individual variation, the 0.253 μM concentration gave only a tendency to increase the CFE. So, we go further in low concentration and selected this concentration of 30 nM, already used by other teams as a photoprotective agent or to delay replicative senescence of human fibroblasts [18]. A portion of our results highlights the positive effect of Selenium on the proliferative properties of keratinocytes and their capacity to give rise to holoclones in culture and their ability to enhance their replicative life span in a monolayer culture. First, we observed that supplemen-tation of keratinocytes during culture enhances the proportion of holoclones. Holoclones in culture represent the KSC population. KSCs are quiescent or low-cycling cells that ensure epidermal renewal over the lifetime. The high interfollicular KSCs anchorage to the basement membrane, corresponding to the follicular niche protects them from apoptosis, proliferation, differentiation and oxidative stress [1,25]. Their proliferative capacities can be activated by external stimuli. This niche can be defined as a micro-environment in which growth factors modulate adhesive interactions, cell cycle regulation and inter-cellular signaling. At this level, integrins and their ligands play a key role in cell-cell and cell-extracellular matrix (ECM) communication and the regulation and maintenance of KSC phenotypes [26–28]. Integrin ligands such as laminins and collagens are the major constituents of the human KSC niche [29]. Some studies are based on selecting a human KSC population by using integrins, showing the importance of integrins in the characterization of KSCs [30,31].

Second, we also observed that Selenium increases the replicative life span of primary keratinocytes from various donors of different ages. This confirms that Selenium supplementation is active despite the donors' differences in genetic background, health conditions, nutrition and age. Here, we note that the capacity of keratinocytes to give rise to holoclones is delayed with Selenium supplementation over subculture passages. It has been described in WI-38 fibroblasts that Selenium at 30 nM can delay replicative senescence [18] through ROS production and oxidative stress. In fact, in a Selenium-depleted culture condition, there is an increase in oxidative stress and the ability to induce replicative senescence via p16, p21 and p53 signaling pathways. This effect can be reversed by the addition of Selenium with the consequence a decrease in ROS and oxidative stress and a delay of replicative senescence. We show that Selenium preserves the keratinocyte stem cell pool and their regenerative potential against replicative senescence in a monolayer culture condition via activation of the adhesive properties of supple-mented keratinocytes over subculture. In fact, the proportion of keratinocytes adhering to laminin 332 and type IV collagen is improved from 133.1 ± 12.4% to 157.4 ± 22.9%, respectively, regardless of the passage (from p0 to p6). Both of these proteins, ligand of β1 and α6 integrin, are involved in the DEJ of skin and in the follicular KSC niche [32,33]. Therefore, we can suggest that in our model, maintenance of holoclones in a monolayer culture during the replicative life span is due to the activation of keratinocyte adhesion to collagen type IV and laminin 332 due to an enrichment of β1- and α6 integrin-expressing KSCs [27,28].

In this study, we also aimed to be closer to aging using a dermo-epidermal model, which is more physiological-ly accurate than a monolayer culture. Indeed, it led to a pluristratified and differentiated epidermis firmly anchored on a living dermis through a DEJ. Our data confirm the impact of Selenium using our SE model over an extended culture time, mimicking chronological skin aging [24]. Indeed, this 3D model based on extending time of culture recapitulates several bio-chemical and morphological modifications associated with chronological skin aging such as decreased proliferation and differentiation, modifications of DEJ and increased P16INK4a expression which is a good biomarker for cell senescence. Investigating the balance between proliferation and differentiation, we highlight that Selenium has a strong beneficial effect on chronological skin aging. As in normal skin aging, our control SE presents signs of aging with culture, as shown by a decrease in epidermal thickness that is linked to a decrease in proliferating cells, an increase in senescence-associated marker p16INK4a [34-36] and a loss of homeostasis. For the treated SE, the conservation of keratinocyte proliferation allows a thicker epidermis over time and a better differentiation, which is followed by filaggrin expression. Simultaneously, the protein p16INK4a (located in both nuclear and cytoplasmic part of the cells as previously describe [37]) appears to be less expressed when the SEs are protected by Selenium. Selenium pre-treatment may decrease p16INK4a expression by its ability to altering post-translational modifications as DNA methylation. Indeed, selenium has been shown to alter promoter DNA demethylation [38], histone deacetylase [39] and kinase activities [40]. Note that p16INK4a CpG island methylation is associated with its transcriptional silencing [41].

On the other hand, selenium beneficial effect on chronological skin aging might be due to the activating effect of Selenium on the adhesion of keratinocytes to the basement membrane, as shown by the over expression of β1 and α6 integrin in the treated SE. Integrin expression is impaired during skin aging [42], but with Selenium supplementation, the expression pattern is maintained. Our results demonstrate that Selenium protects KSCs by preserving integrins and consequently the capacity for epidermal renewal. All together, these data strongly support our hypothesis that Selenium supplementation acts with a significant protective effect against chronological skin aging. These results are in agreement with those suggesting a close interplay between Selenium and healthy aging [21], since serum Selenium has been related to the longevity of the human population [20].

Altogether, our results suggest that the benefits of Selenium could be due to an enhancement in the adhesive properties of KSCs in a monolayer culture and reconstructed tissue that could allow KSC preservation through replicative senescence and skin aging. We propose that Selenium could be a good “anti-aging” agent and could be used to protect skin from chronological aging and the decrease of keratinocytes stemness of lifetime.

## MATERIALS AND METHODS

### Tissue harvest

Normal human skin tissue explants were obtained from the surgical discard of anonymous healthy patients with informed consent in accordance with ethical guidelines and declared to the French research ministry (Declaration no. DC-2008-162 delivered to the Cell and Tissue Bank of Hospices Civils de Lyon).

### Fibroblast and Keratinocyte cultures

Fibroblasts were grown in Dulbecco's modified Eagle's medium (DMEM) supplemented with 10% fetal calf serum (FCS), antibiotics (20 mg/mL gentamicin (Panpharma, Fougères, France), 100 IU/mL penicillin (Panpharma), and 1 mg/mL amphotericin B (Panpharma). Keratinocytes were grown on a feeder layer of irradiated human fibroblasts pre-seeded at 4000 cell/cm^2^, as previously described [23,43], in a mixture of 3:1 DMEM and Ham's F12 (Invitrogen, Carlsbad, USA), respectively, supplemented with 10% FCS, 10 ng/mL epidermal growth factor (EGF; R&D systems, Minneapolis, MN, USA), 0.12 IU/mL insulin (Lilly, Saint-Cloud, France), 0.4 mg/mL hydrocortisone (UpJohn, St Quentin en Yvelines, France), 5 mg/mL triiodo-L-thyronine (Sigma, St Quentin Fallavier, France), 24.3 mg/mL adenine (Sigma), isoproterenol (Isuprel, Hospira France, Meudon, France) and antibiotics as above. For each cell type, the medium was changed every 2 days until confluence was reached. At confluency, cells were re-suspended with trypsin-EDTA 0.05% (Thermo Fisher Scientific, Waltham, MA USA) and used for the in vitro 3D model or the colony forming unit and toxicity assays.

### Cytotoxicity assay

Keratinocytes or fibroblasts were seeded in 6 -well plates and grown until 80% confluency. Then, NaSe, provide by Labcatal Pharmaceuticals as pharmaceutical liquid solution in sterile water, was diluted in keratinocyte or fibroblast cultures at a concentration ranging from 63.3 μM to 0.0633 μM for 72 h. After incubation, a solution of tetrazolium dye MTT (3-(4,5-dimethylthiazol-2-yl)-2,5-diphenyltetrazolium bromide) in phosphate buffered saline (PBS) at 1 mg/ml was added to the supernatant of the cells for 2 h. The formazan precipitate was solubilized using dimethyl sulfoxide and transferred to 96-well plates to measure absorbance at 560 nm using a spectrophotometer. For each concentration, 3 wells and 12 measurements per well were obtained for 3 independent donors. Viability is calculated as the “(mean of treated cells–mean of blank) / (mean of untreated cells–mean of blank)” ±SD (n=3 donors and n=12 measurements per conditions). The stars illustrate statistical differences between untreated condition (100%) and mean of experimental points.

### Clonogenic assay

Colony forming efficiency (CFE) was evaluated with a large-scale treatment of low-dose NaSe at concentrations ranging from 6.3 μM to 1.875 nM. Keratinocytes were seeded at clonal density, 10 to 20 cells/cm^2^, onto a feeder layer and cultivated for 10 to 14 days. Three flasks were fixed and colored using rhodamine B (Sigma) at 0.01 g/mL in 4% para-formaldehyde for 30 min. The CFE was calculated according to Barrandon et al. [43] as CFE = CFU (meroclones + holoclones number) × 100)/(cellular seeding density). In our manuscript the term “holoclone” was used to refer to large (≥5 mm) and homogeneous clones with regular and smooth contours whereas meroclones are intermediate sized, hetero-geneous and display contours. For the populating doubling calculation, cells from 3 flasks were re-suspended by a trypsin treatment and used for cell numeration. Population doubling was determined as PD = ln (number of harvested cells/number of cells seeded)/ln2 (with ln for natural logarithm). For the analysis of the extended life span, cells from each donor were separated into two groups: treated (KT) or control (KC). The supplementations began at the seeding of the primary culture (just after keratinocytes extraction) and persisted throughout along passages. CFU/CFE was performed at each passage both for KC and KT. The experiments were performed on six independent donors: 3 for the low-dose scale of NaSe and 3 for the extended life span (n=6 flasks for each condition).

### Adhesion assay

Because KSCs are characterized by their strong adhesion to the ECM in “niches” through β1 and α6 integrins [28], we tested the keratinocyte adhesion to their respective ligands, laminin 332 and type IV collagen, the main components of DEJs [44]. For that, multi-well tissue culture plates (Costar, Dutscher, Brumath, France) were coated with the indicated concentrations of laminin 332 [45] or collagen IV (Thermo Fisher Scientific, Waltham, MA, USA) substrates by overnight adsorption at 4°C. Cells were detached by incubation with trypsin and rinsed in serum-free medium prior to the experiment. After saturating the wells with 1% BSA, the plates were used immediately for cell adhesion assays (30 000 cells/well) in serum-free medium containing selenium or not at the concentration used for cell culture. The same amount of cells was also plated on poly-D-lysine coated plates (Biocoat Cell Environments, 6 wells per condition) to evaluate the maximal cell attachment capacity as previously described [46]. After 30 min to 1 h, non-adherent cells were washed with PBS, and the extent of adhesion was determined after fixation of adherent cells, followed by staining with 0.1% crystal violet and absorbance measurements at 570 nm. A blank value corresponding to BSA-coated wells was subtracted. In all experiments, each assay point was derived from triplicate measurements (three wells per assay point) and each adhesion score was normalized to the mean of adhesion obtained from poly-D-lysine coated plates.

### In vitro 3D human skin equivalent (SE) culture

SE cultures were prepared as described previously [23]; fibroblasts from young donors were seeded at a final density of 250 000 cells/cm2 onto a dermal substrate made of chitosan-cross-linked collagen-glycosamino-glycan matrix [47]. This SE was grown in fibroblast medium supplemented with 50 μg/mL L-ascorbic acid (Sigma) and 10 ng/ml of EGF at 37°C in a 5% CO2 atmosphere, and the medium was changed every day for 21 days. For the preparation of SE from keratinocytes, keratinocytes from young donor were seeded onto the SE on day 21. These submerged SEs were cultured for 7 days in keratinocyte medium and then raised at the air-liquid interface and cultured in a simplified keratinocyte medium containing DMEM supplemented with 10% FCS, 10 ng/mL EGF, 0.12 IU/mL insulin, 0.4 mg/mL hydrocortisone, and antibiotics for 17 days or 32 days. The NaSe was added extemporaneously to the culture medium for a final concentration of 30 nM. Samples were systematically harvested after 45 and 60 days of total cell culture and were immediately fixed in neutral buffered formalin 4% (Alphapath, Mudaison, France) for 24 h then embedded in paraffin or directly in OCT compound (Euromedex, Strasbourg, France). For each cell culture condition and analysis, SEs were produced in triplicate.

### Histology and immunohistological (IH) analyses

Paraffin-embedded formalin-fixed samples were cut into 5-μm sections. After dewaxing and rehydration, sections were stained with hematoxylin phloxine saffron (HPS staining). For immunohistochemistry, after heat-mediated antigen retrieval treatment, tissue sections were incubated in 5% H_2_O_2_/3% normal goat serum (NGS; Jackson Immunoresearch, Suffolk, UK) to inactivate endogenous peroxidases. Non-specific binding was blocked in PBS containing 4% bovine serum albumin (BSA, Sigma, St Quentin Fallavier, France) and 5% NGS. Sections were then incubated with the following primary antibodies (Table 1) diluted in PBS/4% BSA/5% NGS overnight at 4°C.

**Table 1:** Primary antibodies

Clone	Class	Specificity	Origin	Dilution Used
**MIB-1**	Mouse IgG1	Ki-67	DakoCytomation, Glostrup, Denmark	1:50
**LKh15**	Mouse IgG2a	Cytokeratin 15	Thermo Fisher Scientific, Waltham, MA, USA	1:200
**E6H4**	Mouse IgG2a	P16INK4A	Ventana Medical systems, USA	
**15C10**	Mouse IgG1	Filaggrin	Novocastra Laboratories	1:25
**P5D2**	Mouse IgG1	Integrin β1	Santa Cruz Biotechnology, Inc, Heidelberg, Germany	1:500
**NIK-GoH3**	Rat IgG2a	Integrin α6	Millipore	1:500

After incubation for 1 h with a peroxidase-conjugated secondary antibody (EnVision, Dakocytomation, Glostrup, Denmark), the antigen was detected with 3,3-diamino-benzidine tetrahydrochloride as the substrate. Tissue sections were subsequently counterstained using Harris hematoxylin (25%, Sigma). For immuno-fluorescence, labeling was performed on air-dried 5-μm cryosections. Air-dried cryosections were incubated with mono-clonal-mouse primary antibodies overnight at 4°C. Then, sections were incubated with secondary Alexa 488- or 568-conjugated anti-mouse or anti-rabbit anti-bodies (Molecular Probes, Invitrogen, Carlsbad, USA) for 1 h at room temperature. Nuclear counter-staining using Hoechst stain was carried out according to a routine protocol. Image acquisition was performed using an Eclipse 50i microscope (Nikon, Champigny sur Marne, France) for light microscopy and a Zeiss LSM 510 confocal laser scanning microscope for fluorescence.

Antibodies used and operating conditions are displayed in Table 1.

### Image analysis and processing

Image processing and analysis were performed using the software Image J with the MBF plugin for microscopy (http://www.macbiophotonics.ca/imagej/, Research Service Branch, US National Institute of Health, United States).

The living epidermal thickness, consider as all layers from basal layer to stratum granulosum excepted the stratum corneum, was measured as the distance between the basement membrane to the stratum corneum and expressed in μm on at least 20 fields per sample. Ki67 and P16INK4a epidermal positive cells were automatically separated from the background and, counted. Data are expressed in number of positive cells per epidermal field.

The results are express as the surface area of filaggrin, and the relative expression for cytokeratin 15, integrin α6 and β1. Positively stained-tissue areas were automatically detected and segmented from other pixels and automatically measured. Data were normalized by the area of living epidermis for filaggrin expression, and by the length of basement membrane for cytokeratin 15, integrin α6 and β1. Measurements are performed on at least 3 different fields of 3 different SE per experimen-tal condition.

### Statistical analysis

For all data, the statistical significance was assessed using Mann Whitney test with the software GraphPad Prism 4 (GraphPad Software Inc., La Jolla, CA, USA), and statistically significant differences are indicated by asterisks as follows: *p<0.05, **p<0.01 and ***p<0.001 (or otherwise indicated on the figure, depending on the experiment).

## Abbreviations

CFE: colony forming efficiency
CFU: colony forming unit
CPD: cumulative population doubling
ECM: extra cellular matrix
EDTA: Ethylenediaminetetraacetic acid
KSCs: keratinocyte stem cells
NaSe: sodium selenite
OCT: optimum cutting temperature
PBS: phosphate buffered saline
PD: population doubling
SE: skin equivalent
